# An approach of molecular-fingerprint prediction implementing a GAT

**DOI:** 10.1039/d5ra00973a

**Published:** 2025-04-22

**Authors:** Chengzhi Deng, Chengli Zhou, Lei Shi, Bingyi Wang

**Affiliations:** a Institute of Highland Forest Science, Chinese Academy of Forestry Kunming 650233 Yunnan P. R. China leishi@139.com; b Nanjing Forestry University Nanjing 210037 Jiangsu P. R. China; c Key Laboratory of Smart Drugs Control of Ministry of Education, Yunnan Police College Kunming 650223 Yunnan P. R. China wbykm@aliyun.com

## Abstract

In the domain of metabolomics, the accurate identification of compounds is paramount. However, this process is hindered by the vast number of metabolites, which poses a significant challenge. In this study, a novel approach to compound identification is proposed, namely a molecular-fingerprint prediction method based on the graph attention network (GAT) model. The method involves the processing of fragmentation-tree data derived from tandem mass spectrometry (MS/MS) data computation and the subsequent processing of fragmentation-tree graph data with a technique inspired by natural language processing. The model is then trained using a 3-layer GAT model and a 2-layer linear layer. The results demonstrate the method’s efficacy in molecular-fingerprint prediction, with the prediction of molecular fingerprints from MS/MS spectra exhibiting a high degree of accuracy. Firstly, this model achieves excellent performance in receiver operating characteristic (ROC) and precision–recall curves. The factors that have the most influence on the resultant performance are identified as edge features using different training parameters. Then, better performance is achieved for accuracy and *F*_1_ score in comparison with MetFID. Secondly, the model performance was validated by querying the molecular libraries through methods commonly used in related studies. In the results based on precursor mass querying, the proposed model achieves comparable performance with CFM-ID; in the results based on molecular formula querying, the model achieves better performance than MetFID. This study demonstrates the potential of the GAT model for compound identification tasks and provides directions for further research.

## Abstract 2

In this study, a novel approach to compound identification is proposed, namely a molecular-fingerprint prediction method based on the graph attention network (GAT) model.

## Graphical abstract

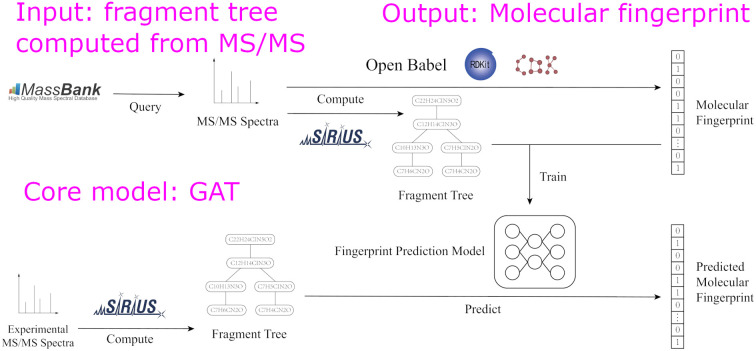

## 1 Introduction

Metabolomics is defined as the unbiased, global survey of all small molecules or metabolites present in a biofluid, cell, tissue, organ, or organism.^1^ As the most downstream of multi-omics process of genomics, transcriptomics and proteomics, metabolomics plays an important role in many fields, including biotechnology, biomedicine and pharmaceuticals.^2^ Presently, the quantity and accuracy of metabolites that can be identified are the key factors restricting the application of metabolomics. Consequently, enhancing the precision of metabolite identification holds promise for optimizing the efficacy of metabolomics analysis.

Mass spectrometry (MS) has emerged as a pivotal instrument in metabolite identification, facilitating comprehensive metabolomics analyses. This technique boasts several key advantages, including high sensitivity and specificity, along with the ability to analyze minimal sample volumes, rendering it a highly efficient tool for metabolite profiling.^3^ Nevertheless, MS furnishes a paucity of data regarding the elemental compositions and chemical structures of fragments.^2^ Tandem mass spectrometry (MS/MS) is a widely utilized technique that facilitates the acquisition of additional information regarding the chemical structures of compounds.^4^ The identification of metabolites in MS or MS/MS spectra constitutes a pivotal step in the subsequent chemical biology interpretation and modelling of metabolomics samples. In practice, this process is regarded as the most challenging and time-consuming aspect of metabolomics experiments. The fragmentation of metabolites, in contrast to the relatively straightforward process of peptide and protein fragmentation due to structural repetitions, is a more intricate and probabilistic process, characterized by the presence of different fragmentation energies. Consequently, the interpretation of mass spectra demands specialized knowledge and expertise. To address this need, numerous computational techniques and software tools have been developed to facilitate metabolite identification in metabolomics experiments.

Computational techniques for metabolite identification can generally be divided into four categories:^2^ (1) mass spectrum libraries: the MS/MS spectrum of the unidentified compound is compared with the reference compound spectra in the mass spectrometry database,^4,5^ and the candidates are scored and ranked according to their similarity to the queried spectrum. The commonly used databases are METLIN,^6^ HMDB,^7^ MassBank,^8^ GNPS,^9^ PubChem,^10^ KEGG,^11^*etc.* Nevertheless, the extent of metabolite coverage provided by these databases is considerably limited in comparison to the substantial quantity of metabolites present in nature. Consequently, their capacity to identify unknown metabolites is somewhat constrained.^12^ (2) *In silico* fragmentation: a software tool for predicting fragments and their abundance from the molecular structure of compounds to fill the gap between spectral and structural databases. This strategy has been successfully applied to protein research (*e.g.*, MASCOT^13^ and SEQUESTEng^14^). In contrast, the fragmentation of product ions of metabolites in MS/MS is a much more complicated stochastic process, depending on the 3D structures of the metabolites, the energy required to break specific bonds to obtain the product ions, the probability of different dissociation reactions, *etc.*^2^ (3) Fragmentation trees: Böcker and Rasche^15^ proposed the use of fragmentation trees for interpreting MS/MS spectra. Fragmentation trees can provide several benefits, such as being used to identify the molecular formula of a molecule, and being used to interpret the fragmentation process of a precursor ion through MS/MS spectra.^16^ In addition, it can be used for comparison by aligning fragmentation trees of two unknown compounds, which can lead to the introduction of useful information about compounds that cannot be identified, such as clustering.^17,18^ (4) Machine learning: in recent years, several machine-learning frameworks have been used to tackle metabolite identification tasks. For example, Brouard *et al.*,^19^ Dührkop *et al.*,^20^ and Heinonen *et al.*^21^ have proposed several methods for predicting substructures or general chemical properties. Mrzic *et al.*^22^ and van der Hooft *et al.*^23^ proposed a method for automatic discovery of substructures from MS/MS spectra, and then identifying candidate compounds from databases based on their substructures.

Molecular fingerprinting is a method of encoding the structure of a molecule that can be converted into a bit string. Each bit in this bit string represents the presence or absence of a substructure in the molecule. This method has applications in the comparison of molecular similarity and the identification of molecules with matching substructures. A multitude of molecular fingerprinting algorithms have been developed, including Avalon, MACCS, Morgan and Klekota–Roth, among others. A variety of instruments are available for the purpose of calculating molecular fingerprints, including: Open Babel,^24^ RDKit,^25^ CDK^26^ and so on. Meanwhile, MetFID,^27^ FingerID,^21^ CSI:FingerID^20^ and many other tools have been developed for predicting molecular fingerprints from MS/MS spectra.

**Fig. 1:**
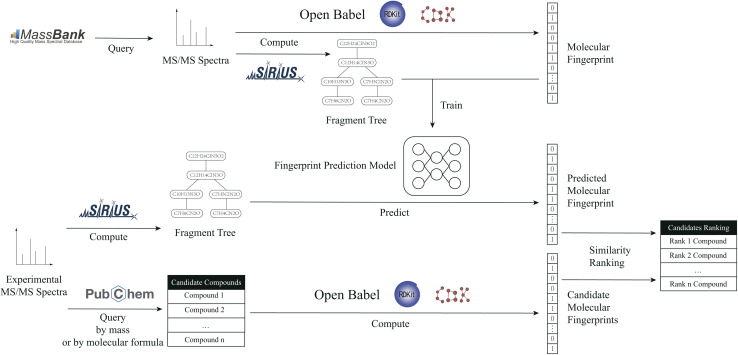
The workflow of the proposed method.

In this study, a methodology is proposed for the prediction of molecular fingerprints of compounds from fragmentation-tree data, which are calculated from MS/MS spectra. The method can improve the accuracy of molecular-fingerprint prediction and help better metabolite identification. The workflow is shown in Fig. 1.

## 2 Data processing and modelling

### 2.1 Graph attention network

A graph attention network (GAT) is a type of graph neural network (GNN) proposed by Veličković *et al.*^28^ that can learn the representation of nodes in a graph. The GAT model is predicated on the attention mechanism, which has the capacity to assign varying weights to disparate nodes in the graph. The GAT model has been demonstrated to facilitate the learning of the representation of nodes in a graph, as well as the prediction of the properties of nodes in the graph. The GAT model has been extensively applied in various domains, including social network analysis, recommendation systems, and bioinformatics.

For a given graph *G* = (*V*, *E*), *V* represents the set of vertices (or nodes) in the graph and *E* represents the set of edges (or connections) between the vertices. Each node *v*_*i*_ ∈ *V* has a feature vector 
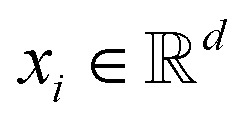
, where *d* is the dimension of the feature vector. The GAT model can be defined as follows:1
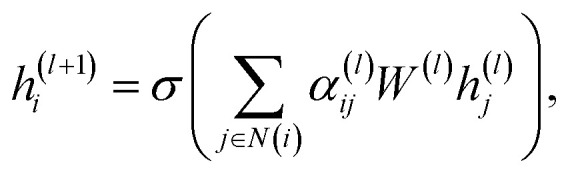
where *h*^(*l*)^_*i*_ is the representation of node *v*_*i*_ at layer *l*, *σ* is the activation function, *N*(*i*) is the set of neighbors of node *v*_*i*_, *W*^(*l*)^ is the weight matrix at layer *l*, and *α*^(*l*)^_*ij*_ is the attention weight between node *v*_*i*_ and node *v*_*j*_ at layer *l*. The attention weight *α*^(*l*)^_*ij*_ can be calculated as follows:2
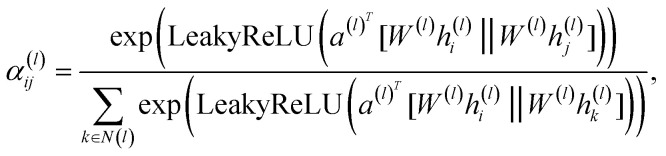
where *a*^(*l*)^ is the attention weight vector at layer *l*, *T* represents transposition, ‖ is the concatenation operation, and LeakyReLU is the activation function. The attention weight *α*^(*l*)^_*ij*_ is calculated based on the feature vectors of node *v*_*i*_ and node *v*_*j*_ at layer *l*. The GAT model can utilize the multi-head attention mechanism to enhance the representation of nodes in the graph. The final representation of node *v*_*i*_ can be concatenated (eqn (3)) or averaged (eqn (4)) as follows:3
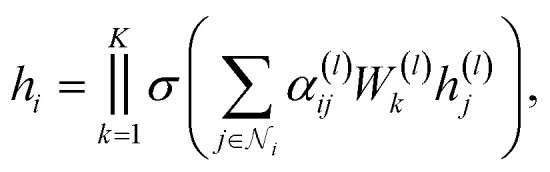
4
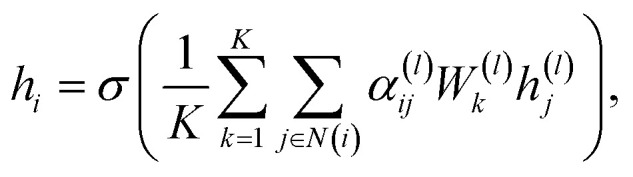
where *K* is the number of attention heads, *W*^(*l*)^_*k*_ is the weight matrix of attention head *k*, and *h*_*i*_ is the final representation of node *v*_*i*_.

### 2.2 Dataset processing

The data utilized in this study were obtained from MassBank and were released in September 2023. The database contains a total of 96 449 entries, of which 75 067 have been found to include MS/MS data. These data are derived from 20 442 compounds. The SIRIUS^29^ software was employed to generate the fragmentation-tree data from the MS/MS data. A total of 52 548 entries were successfully processed.

The fragmentation-tree data were transformed into a graph data structure. Each node in the graph corresponds to a specific fragment, with the molecular formula (encoded using one-hot encoding) and relative abundance of the fragment being represented within the feature vector. Each edge in the graph represents the relationship between two fragments, with the feature vector of each edge being calculated based on the approach of Yao *et al.*,^30^ which is usually used in natural language processing.

For two nodes *i* and *j* in the graph, the feature vector of the edge between them can be calculated as follows:5
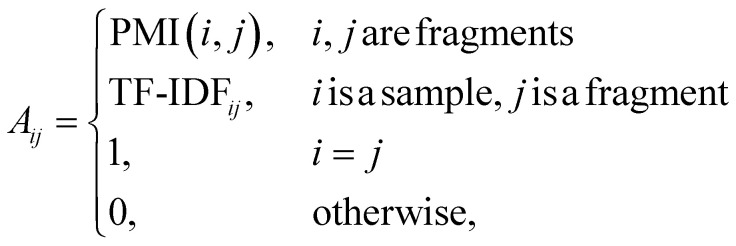
where PMI(*i*, *j*) is the pointwise mutual information of fragment *i* and fragment *j*, TF-IDF_*ij*_ is the term frequency-inverse document frequency between sample *i* and fragment *j*. Both PMI and TF-IDF are derived from the field of information retrieval; PMI is a statistical method used to measure the degree of association between two events,^31^ and TF-IDF is a statistical method used to measure the importance of a word in a text.^32^ PMI is calculated as follows:6a
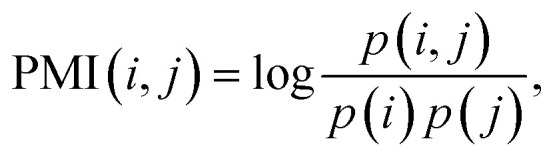
6b
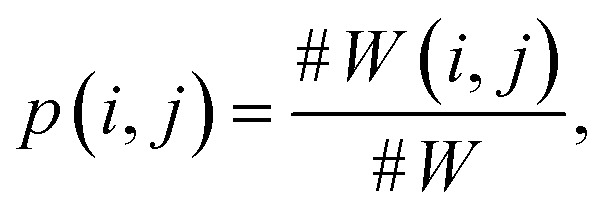
6c
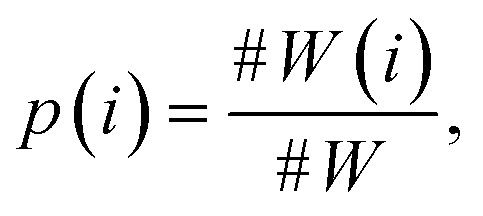
where *p*(*i*, *j*) in (eqn (6a)) is caculated using (eqn (6b)), *p*(*i*) and *p*(*j*) in (eqn (6a)) are caculated using (eqn (6c)), *#W*(*i*, *j*) is the number of times fragment *i* and fragment *j* appear in the same edge, *#W*(*i*) is the number of times fragment *i* appears in an edge, and *#W* is the total number of edges. TF-IDF is calculated as follows:

7a TF-IDF_*ij*_ = TF_*ij*_ × IDF_*j*_,

7b
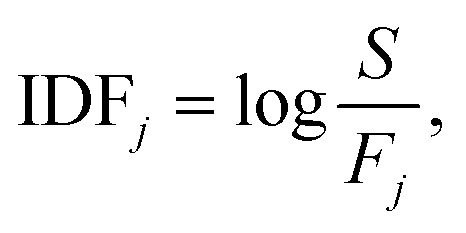
where TF_*ij*_ is the term frequency of fragment *j* in sample *i* (here we use relative intensity to represent the term frequency), IDF_*j*_ is the inverse document frequency of fragment *j*, *S* is the total number of samples, and *F*_*j*_ is the number of samples that contain fragment *j*.

A total of 16 659 bits of molecular-fingerprint data were generated based on the molecular structure information (from SMILES or InChI) of the compounds. Among them, 1024 bits of FP2 fingerprints were generated using Open Babel; 2048 bits of Atom Pair fingerprints, 512 bits of Avalon fingerprints, 166 bits of MACCS fingerprints, 2048 bits of Morgan fingerprints, and 2048 bits of RDKit fingerprints were generated using RDKit; and 1024 bits of CDK fingerprints, 881 bits of PubChem fingerprints, and 4860 bits of Klekota–Roth fingerprints were generated using CDK.

The dataset was constructed by taking the graph data (including node features, edges, and edge features) as input values and the molecular fingerprints as output values.

To ensure the robustness of the dataset, a ten-fold cross-validation method was employed to divide the dataset into ten copies. In each iteration, nine of the ten copies were designated for training, while the remaining one was allocated for testing. A total of 10 training and testing sessions were conducted, and the average of the 10 tests was finally obtained as the final result.

### 2.3 Modelling

**Fig. 2:**
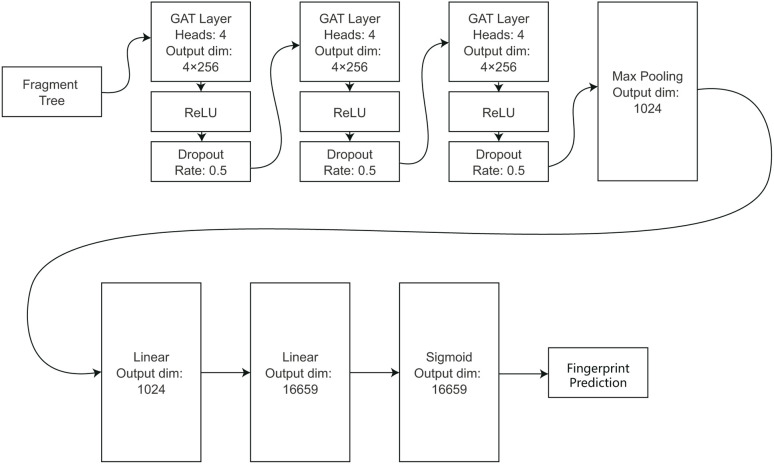
The structure of the model.

The structure of the model is shown in Fig. 2. The model’s core comprises three layers of GAT: layer 1 accepts the graph data as input, and layers 2 and 3 accept the output of the previous layer. The number of attention heads in each GAT layer is 4, and the output dimension of each head is 256. The total output dimension of each layer is 1024. The activation function employed is ReLU. A dropout policy is implemented to prevent overfitting, with a dropout rate of 0.5.

Subsequent to the GAT layer is a pooling layer that utilizes a max-pooling strategy to convert the feature vector of each node into a scalar. Subsequently, the data undergoes two linear fully connected layers, followed by a sigmoid function that transforms the output value into a range between 0 and 1. This value is then employed as the predicted value of the molecular fingerprint.

The training process was optimized through the implementation of batch gradient descent, employing a batch size of 64. The loss function was defined as binary cross-entropy loss (BCELoss). The Adam with decoupled weight decay (AdamW)^33^ optimizer was employed. Following a preliminary evaluation, a learning rate of 0.0001 and a weight decay of 0.0001 were identified as the optimal parameters.

In order to verify the factors that have the most influence on the model, the model was trained using different strategies. These include: training more epochs, using a different size of datasets, deleting node features or edge features from the dataset and scaling down the number of GAT layers or linear layers.

## 3 Results and discussion

### 3.1 Evaluation of training

Following the training process, the following resultant models were obtained: 300 training epochs with the full dataset (FF), 5000 training epochs with the full dataset (FFn), 300 training epochs with the limited dataset (LF), 300 training epochs with a reduced number of linear layers (FL), 300 training epochs with censored edge features (FFe), and 300 training epochs with censored node features (FFv). The full training set consists 47 293 data points, and the test set consists of 5255 data points. The training set for LF consists of 9000 data points, while the test set comprises 1500 data points, akin to the MetFID approach. During the training process, randomly selected data from the training set that is comparable to the test set is used for validation. The linear layer of FL is reduced to a single layer. FFe retains only the edge connectivity, while eliminating the edge weight information. FFv retains only the relative abundance of node information, while deleting the elemental information and the mass-to-charge ratio information. It was observed that the models with a reduced number of GAT layers were not adequately trained and failed to complete the training process.

**Fig. 3:**
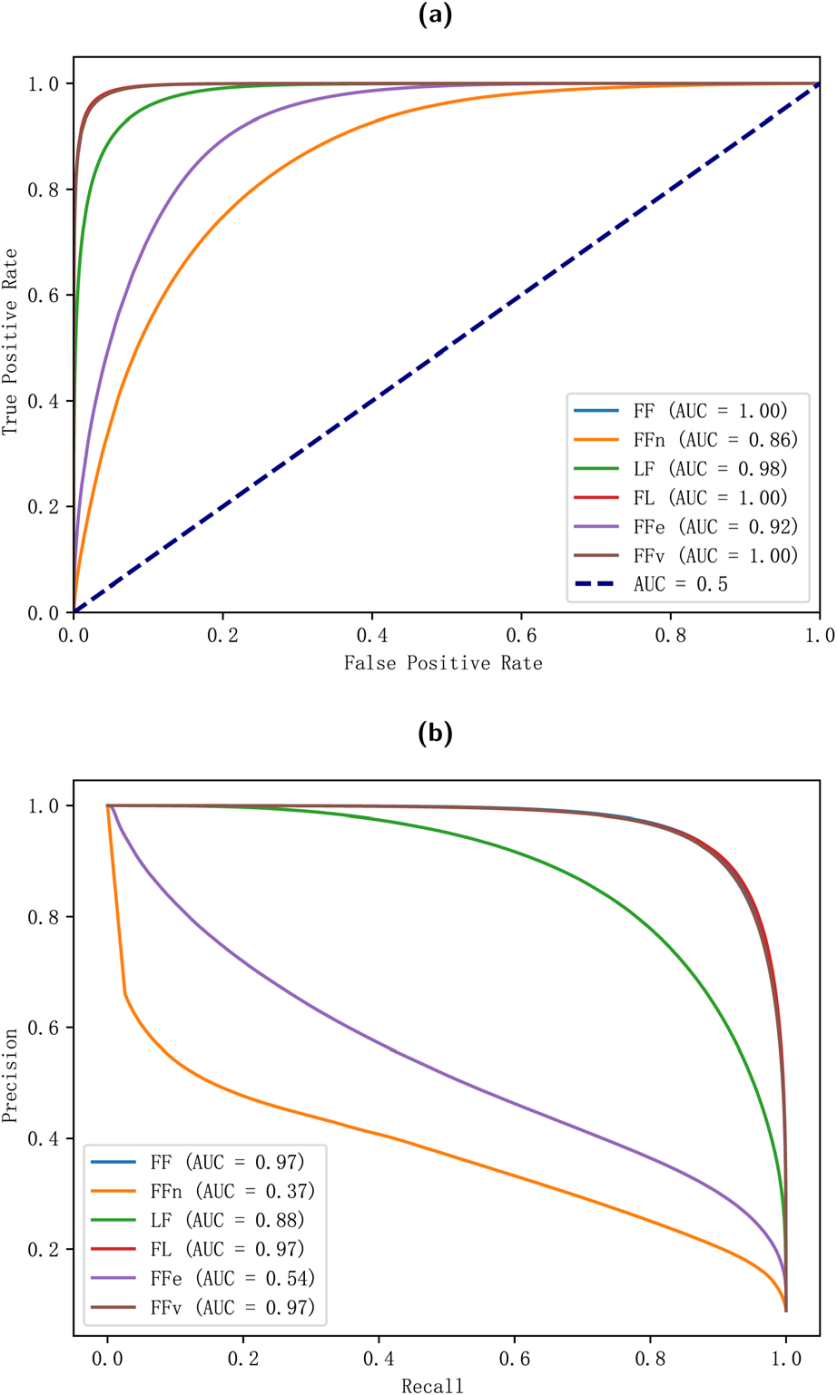
The (a) ROC and (b) precision–recall curves of the models.

The receiver operating characteristic (ROC) curve for each model is shown in Fig. 3a and the precision–recall curve is shown in Fig. 3b. A ROC curve is a graphical representation of the true positive rate (sensitivity) against the false positive rate (1 − specificity) for different cut-off points of a diagnostic test. A precision–recall curve is a graphical representation of the precision against the recall for different cut-off points of a classification model. The area under curve (AUC) of the ROC curve is a measure of the model’s ability to distinguish between the positive and negative classes and ranges from 0 to 1, with 1 indicating perfect discrimination and 0.5 indicating no discrimination (equivalent to random guessing). The AUC of the precision–recall curve is a measure of the model’s ability to identify positive samples and ranges from 0 to 1, with 1 indicating perfect identification and 0 indicating no identification.

The FF, FL and FFv models demonstrate optimal performance, exhibiting an AUC of 1.0 in the ROC curve and 0.97 in the precision–recall curve. These results indicate the models’ capacity to accurately differentiate between positive and negative samples and their high precision across a range of recall levels. This suggests that the models excel not only in identifying positive instances but also in doing so with a high degree of accuracy. The LF model demonstrates notable efficacy with an AUC of 0.98 in the ROC curve and 0.88 in the precision–recall curve, indicating robust performance. The FFe and FFn models also demonstrate adequate performance with AUCs of 0.92 and 0.86 in the ROC curve, respectively. However, these models exhibit suboptimal performance with AUCs of 0.54 and 0.37 in the precision–recall curve, indicating that their discrimination capabilities are satisfactory but not exceptional. Consequently, the trade-offs between precision and recall are less effective, resulting in a lower overall performance compared to the other models.

The findings indicate that the elemental information and the mass-to-charge ratio information exert a negligible influence on the model’s performance. Conversely, the relative abundance of node information emerges as the paramount factor contributing to the model’s efficacy. The edge weight information emerges as the most significant factor contributing to the model’s performance. The number of GAT layers is also found to be of significant importance, with a lack of layers potentially hindering the model’s ability to complete training. In contrast, the impact of linear layers is relatively minimal. Notably, the model demonstrates a capacity to attain satisfactory performance even with a reduced volume of training data. Conversely, an augmentation in the number of training epochs has a substantial adverse effect on the model’s performance, signifying an overfitting problem.

To demonstrate our models’ superiority, we compared the performance of our models with MetFID.^27^ The MetFID model is a machine learning model that can predict molecular fingerprints from MS/MS data. The results for comparison were obtained from a MetFID model trained using spectra with all collision energies, and tested on separated datasets with collision energies less than 30 eV and greater than 30 eV; and from another one trained using combined spectra generated by ion-trap (IT) and higher-energy collisional dissociation (HCD) instruments, and tested on separated datasets of IT and HCD instruments. The accuracy and *F*_1_ score of the MetFID model and our models are shown in Table 1.

**Table 1:** Accuracy and *F*_1_ score of different models

Model	Data set	Accuracy	*F*_1_ score
MetFID	(⩽30 eV)	92%	58%
	(⩾30 eV)	94%	69%
	(IT)	94%	74%
	(HCD)	94%	68%
Proposed models	FF	97.7%	85.6%
	FFn	98.8%	93.3%
	LF	95.8%	71.7%

Compared to MetFID models with different data sets of the same size, the LF model achieves an accuracy advantage of 1.8 to 3.8 percentage points (pp) and an *F*_1_ score difference of −2.3 to 13.7 pp. This demonstrates the advantages of this model over MetFID. For the FF model using a larger data size, there is a further improvement in accuracy of 1.9 pp and an improvement in *F*_1_ score of 13.9 pp compared to the LF model, indicating that this model is able to achieve a significant improvement on larger data sets. The FFn model with an increased number of training epochs shows a further improvement in accuracy of 1.1 pp and an improvement in *F*_1_ score of 7.7 pp compared to the FF model, indicating that the number of training epochs also has a significant effect on model effectiveness.

The FFn model has a lower AUC on the PR curve, but still achieves better results in terms of accuracy and *F*_1_ scores, which is due to the ROC curve being relatively robust to the imbalance of positive and negative samples, and the PR curve reflects the model’s ability to predict positive samples, whereas the accuracy and *F*_1_ scores reflect the model’s ability to predict all samples (including positive and negative samples). In the molecular-fingerprint prediction task, positive samples indicate the presence of substructure and negative samples indicate the absence of substructure. For a given molecule, there are only a limited number of substructures and most are absent, so the number of negative samples is much higher than the number of positive samples.

### 3.2 Evaluation of prediction

In order to evaluate the models’ prediction effect, this study employed the molecular library query method that has been widely utilized in related studies. Specifically, a molecular library was constructed, and the candidate compounds were retrieved from the molecular library by using the precursor mass and molecular formula (both of which are available in the MS/MS data) as the query conditions. To query the precursor mass, the exact mass in the MS data was used. Due to the inherent error in precursor mass, a precision window (5 ppm, 20 ppm, 50 ppm, or 100 ppm) was established when querying the database. Subsequently, the molecular fingerprints predicted by the models of this study were compared with those of the candidate compounds, and scored and ranked. The proportion of correct molecules containing samples among the candidate compounds that were located in the top 1, top 5, and top 10 in the ranking was finally calculated.

The compound data for the construction of the molecular libraries were obtained from the PubChem database, a free database created and maintained by the National Library of Medicine (NLM) that contains information on more than 100 million compounds. The molecular formula, molecular weight, SMILES, InChI, and other pertinent information of the compounds are included in the PubChem database.

A multitude of similarity-score methods can be utilized to compare the molecular fingerprints of the predicted compounds and the candidate compounds. These include the Tanimoto coefficient, the Dice coefficient, the Cosine coefficient, and the Euclidean distance. In this study, the Cosine coefficient was selected due to its effectiveness in the preliminary experiment. The Cosine similarity score is calculated as follows:8
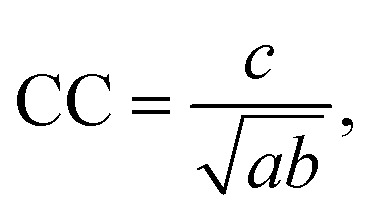
where *a* is the number of bits of value 1 in the predicted bits, *b* is the number of bits of value 1 in the true bits, and *c* is the number of bits of value 1 in both the predicted bits and the true bits.

The results based on precursor mass querying are shown in Table 2. The results of CFM-ID, MetFrag, and FingerID are collected from Allen *et al.*^34^ These were trained using MassBank data and tested by querying the PubChem database. The FF model’s performance, particularly within the 5 ppm window, exhibits a noticeable discrepancy compared to that of CFM-ID. This disparity can be attributed to the incomplete consistency of the molecular libraries utilized. However, the performance of the results exhibits a substantial enhancement upon further training of the FFn model. The LF model’s page table entries for the limited training set demonstrate comparable performance, indicating the model’s strong generalisation capability. All models proposed in this study show a competitive performance over other models.

**Table 2:** Results based on precursor mass querying

Model	Accuracy	Top 1	Top 5	Top 10
CFM-ID	5 ppm	7.3%	—	46.9%
MetFrag	5 ppm	4.7%	—	20.8%
FingerID	5 ppm	0.5%	—	5.7%
FF	5 ppm	5.1%	6.1%	6.3%
	20 ppm	7.6%	9.5%	9.9%
	50 ppm	7.4%	9.7%	10.2%
	100 ppm	7.8%	10.2%	10.6%
FFn	5 ppm	9.1%	9.9%	10.1%
	20 ppm	9.5%	10.5%	10.7%
	50 ppm	9.9%	11.0%	11.3%
	100 ppm	10.3%	11.3%	11.7%
LF	5 ppm	4.0%	5.7%	6.3%
	20 ppm	5.5%	8.9%	10.1%
	50 ppm	5.0%	8.3%	9.5%
	100 ppm	4.1%	7.2%	8.3%

The observed discrepancy between the top 5 and top 10 results can be attributed to the presence of inaccuracies in the measured precursor mass values derived from mass spectrometry data. A significant proportion of the sample compounds did not contain the intended molecules when the molecular library query was executed. However, when the correct candidate molecules are incorporated, the model proposed in this study can yield more accurate results. The minor disparity between the top 1 and top 5, as well as the top 10, indicates that the majority of the correct candidate molecules are positioned higher in the ranking.

The observed discrepancy in the outcomes across varying precision ranges can be attributed to the substantial size of the PubChem database. As the precision range is increased, the number of candidate compounds increases considerably. However, the correctly matched candidate compounds are already contained within the smaller precision range. Consequently, the results for the larger precision ranges do not exhibit significant improvement.

The results based on precursor mass querying are shown in Table 3. The results of MetFrag, CSI:FingerID and MetFID are collected from Fan *et al.*^27^ The results of the FF and FFn models for the top 1 show a very good performance lead compared to the comparison models. Although the LF model performs poorly compared to the other models, the results still illustrate that the model proposed in this study can still perform even if the number of training sets is limited, further illustrating the model’s ability to generalise.

**Table 3:** Results based on molecular formula querying

Model	Top 1	Top 5	Top 10
MetFrag	12%	—	—
CSI:FingerID (2016)	28%	55%	70%
CSI:FingerID (2019)	39%	—	75%
MetFID	38%	72%	72%
FF	43.6%	55.0%	58.0%
FFn	54.0%	61.1%	62.6%
LF	22.8%	37.9%	42.1%

### 3.3 Comparative insights

A synthesis of the aforementioned results indicates that the model proposed in this study exhibits commendable performance, at times surpassing the comparison model in specific aspects. An increase in the number of training instances leads to overfitting in certain instances; however, the model maintains optimal performance in molecular library querying, suggesting a moderate complexity level. Furthermore, the model trained with a constrained training set demonstrates acceptable performance, thereby substantiating the model’s adept generalizability.

In comparison with the CFM-ID model, which utilizes the CFM model, and the MetFID model, which employs an artificial neural network (ANN) model, the GAT model with a multi-attention mechanism, as implemented in this study, and fragmentation-tree data for the prediction of molecular fingerprints demonstrates enhanced performance. The cleavage process of MS/MS exhibits a degree of regularity, manifesting as fragmentation at specific chemical bonds. Consequently, the fragmentation-tree is capable of reflecting structural information with higher precision compared to the use of mass spectrometry data alone. The GAT model’s enhanced ability to prioritize significant nodes within the fragmentation tree, in comparison to the conventional GCN model, ensures a more effective learning of the representation of these nodes. Consequently, the methodology proposed in this study demonstrates superior performance in molecular-fingerprint prediction.

The model proposed in this study has certain limitations, despite its strong performance in several performance indicators. The model’s performance is excessively reliant on the molecular library query results. If the molecular library query results are unsatisfactory, the similarity scoring results performed with the molecular fingerprints predicted by the model are also affected. This is particularly evident in the context of precursor mass-based screening, where the precursor mass, as measured in mass spectrometry data, differs from the exact mass in molecular libraries, which is calculated based on theoretical values. This discrepancy leads to significant variations in the querying process, potentially resulting in the exclusion of correct candidate molecules from the query results. This phenomenon is evident in the screening results based on precursor mass, and other models exhibit a similar low bias in this index. Furthermore, the molecular formulae of the mass spectrometry data are also calculated based on the mass spectrometry data, which is subject to bias but is more effective than the precursor mass. Additionally, this model utilizes the existing definition of molecular fingerprints, and there are overlapping features in different molecular fingerprints, which limits the amount of effective information that can be learnt.

Subsequent iterations of the model can be designed to execute additional tasks in multiple domains to enhance its performance. Initially, there is a potential to utilize a more extensive array of mass spectrometry datasets during the training process, with the objective of refining the model’s performance. Additionally, there is a possibility to optimize the molecular library query to facilitate the investigation and proposal of a more precise screening method for candidate compounds. Furthermore, there is a prospect to transcend the limitations of the prevailing molecular fingerprinting framework, thereby enabling the GAT to autonomously extract features. This approach is expected to yield more efficacious information and elevate the efficacy of compound identification.

## 4 Conclusions

In this study, a molecular-fingerprint prediction model based on a graph attention network is proposed. This model can predict molecular fingerprints based on the fragmentation tree generated in SIRIUS from mass spectrometry data. The model can be trained on different mass spectrometry datasets with a good generalisation ability. In both precursor mass-based queries and molecular-formula-based queries, the model can outperform the currently commonly used mainstream models in terms of Top 1 results. The results of this study suggest the potential of applying the GAT in compound identification.

## Data availability

This study was carried out using publicly available data from MassBank at https://massbank.eu/MassBank/ and PubChem at https://pubchem.ncbi.nlm.nih.gov/. The code used in this study is available at https://github.com/qakcn/MassBank2Dataset with DOI: https://doi.org/10.5281/zenodo.14840585 and https://github.com/qakcn/MassBankML with DOI: https://doi.org/10.5281/zenodo.14840544.

## Author contributions

Chengzhi Deng: data curation, formal analysis, software, writing – original draft. Zhoucheng Li: resources, funding acquisition, supervision. Lei Shi: resources, funding acquisition, supervision. Binyi Wang: conceptualization, methodology, validation, writing – review & editing. All authors have read and approved the final manuscript.

## Conflicts of interest

There are no conflicts to declare.
